# Nuclear Lactate Dehydrogenase A Resists Cardiomyocyte Cell Cycle Arrest Induced by Oxidative Stress

**DOI:** 10.3390/jcdd12070278

**Published:** 2025-07-21

**Authors:** Mengfei Cao, Jie Luo, Kewei Fu, Yao Xu, Yinyu Wang, Junying Duan, Rui Chen, Wei Yuan

**Affiliations:** 1 Department of Cardiology, Affiliated Hospital of Jiangsu University, Zhenjiang 212000, China; 2 Department of Cardiology, Yixing People’s Hospital, Yixing 214200, China

**Keywords:** nuclear lactate dehydrogenase A, cardiomyocyte proliferation, oxidative stress, hypoxia

## Abstract

A sudden increase in ambient oxygen concentration after birth forces the metabolic switch from anaerobic glycolysis to oxidative phosphorylation, which contributes to the rapid decline of cardiomyocyte proliferation. Lactate dehydrogenase A (LDHA), a metabolic enzyme normally localized in the cytoplasm, has been reported to regulate cardiomyocyte proliferation via inducing metabolic reprogramming. Nuclear LDHA has been observed in multiple proliferative cells, whereas the role of LDHA nuclear translocation in cardiomyocyte proliferation remains unresolved. Here we found that the expression of nuclear LDHA was induced both in the infarct area of myocardial infarction (MI) in mice and hypoxic cardiomyocytes in vitro. Mechanically, mild hypoxia prompted metabolic reprogramming which motivated cardiomyocyte proliferation by alleviating reactive oxygen species (ROS), while severe hypoxia coincided with oxidative stress that induced cardiomyocyte cell cycle arrest. Interestingly, LDHA nuclear translocation in cardiomyocytes occurred in response to oxidative stress, and blocking of nuclear LDHA resulted in elevated ROS generation. Collectively, our findings uncover a non-canonical role of nuclear LDHA in maintaining redox balance and resisting cardiomyocyte cell cycle arrest.

## 1. Introduction

The adult mammalian heart maintains a low self-renewal rate of 1% per year, which results in limited regeneration following cardiomyocyte loss [1]. A massive loss of cardiomyocytes caused by myocardial infarction (MI) leads to pathological cardiac remodeling and heart failure [2]. Promoting cardiomyocyte proliferation may be the most promising treatment of heart failure. The neonatal heart exhibits remarkable regenerative capacity after myocardial damage [3]. With the metabolic switch from anaerobic glycolysis to oxidative phosphorylation after birth, the majority of cardiomyocytes permanently exit the cell cycle [4]. A sudden increase in ambient oxygen concentration after birth may be the most important environmental factor triggering the metabolic switch of cardiomyocytes. The reactive oxygen species (ROS), which causes oxidative stress and DNA damage in postnatal cardiomyocytes, derives from aerobic respiration [5]. Exposure to gradual systemic hypoxemia reduces ROS and oxidative DNA damage, thereby inducing the re-entry of adult cardiomyocytes into the cell cycle [6]. However, severe hypoxia compromises the electron transport chain (ETC), and electron leak by the ETC is the major source of free radical production, resulting in cell cycle arrest, apoptosis, or cellular senescence [7,8]. It seems that hypoxia may be both beneficial and harmful to cardiomyocyte proliferation. Compared to absolute anaerobic glycolysis, targeting the cellular redox balance could be a viable strategy for mammalian heart regeneration.

Lactate dehydrogenase A (LDHA), a metabolic enzyme that catalyzes the generation of lactate in the cytoplasm, is essential for maintaining glycolysis and cell proliferation [9,10,11,12]. Cardiomyocyte-specific LDHA knockout neonatal mice exhibited damaged cardiac regeneration, leading to worse cardiac function and a lower survival rate in response to apical resection. Mechanically, metabolic reprogramming triggered by LDHA promoted cardiomyocyte proliferation via an alleviation of ROS and oxidative DNA damage [13]. However, current understanding of LDHA in the heart primarily focuses on its cytoplasmic enzymatic activity during proliferation. Intriguingly, emerging evidence, predominantly from oncology, suggests that LDHA can localize to the nucleus and perform non-canonical functions. Nuclear LDHA has been observed in several cancer types [14], where its ROS-induced translocation enhances antioxidant capacity, facilitating survival and proliferation under oxidative stress [15]. While this highlights a potential link between nuclear LDHA, redox balance, and proliferation in cancer cells, its relevance and function in cardiomyocytes remain entirely unexplored. Critically, it is unknown whether LDHA undergoes nuclear translocation in cardiomyocytes, particularly during proliferation or regeneration, and if so, what functional role it might play. Our data provide initial evidence suggesting a potential non-canonical role for nuclear LDHA in cardiomyocytes, possibly contributing to redox balance maintenance and resistance to cell cycle arrest. Based on these preliminary observations and the precedent of nuclear LDHA function in other proliferative contexts, we hypothesize that stimulating LDHA nuclear translocation might represent a novel, albeit exploratory, strategy worthy of further investigation for promoting mammalian heart regeneration.

## 2. Materials and Methods

### 2.1. Animals and Treatment

C57BL/6 J mice were provided by the Laboratory Animal Center of Jiangsu University (Zhenjiang, China). All animal experiments were approved by the Animal Care and Use Committee of Jiangsu University. For a mouse MI model construction, 8-week-old male mice were anesthetized with isofurane gas, and then fixed on the operating table to fully expose the chest. Cut the skin at the fourth rib of the left chest. Then, separate the subcutaneous tissue and the pectoralis major and pectoralis minor muscle layer by layer. Puncture the 4–5 intercostal space to open the ribs, and squeeze out the heart quickly. The left anterior descending branch was permanently ligated with a 6-0 suture needle, and the heart was then reset into the chest cavity. Finally, suture the skin with a 4-0 suture needle. For mouse euthanasia, the animals were executed via inhaling excess isoflurane. Twenty-four h after MI, mouse serum was collected and applied to LDH concentration detection (Shanghai, China, Beyotime, C0016), then the hearts were quickly removed and frozen for TTC staining (Shanghai, yuanye Bio-Technology, R20618). Seven days after MI, the hearts were thoroughly washed, and then preserved in 4% paraformaldehyde (PFA) for further histologic analysis.

### 2.2. Cell Culture and Treatments

C57BL/6 J mice (1–2 days old, sex not distinguished) were purchased from Jiangsu University Laboratory Animal Center. To obtain primary mouse cardiomyocytes, the ventricles of neonatal mice were collected and washed twice with PBS, then the ventricles were cut into pieces and digested with 0.03% trypsin (Wisent, Montreal, QC, Canada) at 4 °C for 12 h. Next, the cardiac tissue slices were digested with 0.04% collagenase type II (Gibco, New York, NY, USA) at 37 °C repeatedly until the tissue pieces disappeared. The supernates from digestive fluid were collected and neutralized with 10% fetal bovine serum (FBS, Wisent, Montreal, QC, Canada). Finally, all the neutralized liquid was centrifuged at 900 r/min for 5 min, the supernatant was discarded and the cells were inoculated in petri dishes. After being pre-plated for 2 h, the medium, which contained cardiomyocytes, was collected and reinoculated at a suitable density. Cardiomyocytes were cultured in DMEM supplemented with 10% horse serum (Gibco, New York, NY, USA), 5% FBS, and 1% penicillin-streptomycin (Wisent, Montreal, QC, Canada) at 37 °C in a 5% CO_2_ incubator. Herein, a hypoxia incubator chamber (Billups-Rothenberg, MIC-101) was used for hypoxic studies in vitro. 2-Deoxy-D-glucose (2-DG, 5 mM, MCE, HY-13966) is an inhibitor of glucose metabolism and inhibits glycolysis by acting on hexokinase. N-acetylcysteine (NAC, 5 mM, Selleck, Houston, TX, USA, S1623) is an inhibitor of ROS, while Berberine chloride (10 µM, Selleck, S2271) is considered to be a ROS agonist. Importazole (40 µM, Selleck, S8446) is a small molecule inhibitor of the transport receptor importin-β, which can specifically inhibit importin-β mediated nuclear translocation.

### 2.3. Histologic Analysis of the Cardiac Tissue

Paraffifin sections were stained with Masson (Solarbio, Beijing, China, G1340) for the valuation of cardiac fibrosis after MI. Hematoxylin and Eosin (H&E) (Solarbio, G1120) staining was used to assess the infiltration of inflammatory cells. The experiments were performed as previously described [16]. For immunofluorescence (IF) staining, paraffifin sections were dewaxed and dehydrated, then blocked with PBS containing 10% goat serum and 0.25% Triton X-100 for 60 min. Anti-8-OHdG antibody (1:50, Santa, Santa Cruz Biotechnology, Dallas, TX, USA, sc-393871) was applied to assess the degree of oxidative damage in cardiac tissue. Anti-LDHA antibody (1:200, Proteintech, 19987-1-AP) was used to observe the expression and subcellular localization of LDHA. Cardiomyocytes were identified by anti-Cardiac Troponin T (cTnT) antibody (1:400, Abcam, ab8295), while anti-Ki67 antibody (1:250, Abcam, ab16667) was used to suggest that cardiomyocytes re-enter the cell cycle. After incubation, the antibodies were removed and the stained sections were observed with a microscope (Olympus, Tokyo, Japan).

### 2.4. Western Blot Analysis

Total protein samples from primary mouse cardiomyocytes were extracted with RIPA Lysis Buffer, which contains protease and phosphatase inhibitors (New Cell & Molecular Biotech, Newcastle upon Tyne, UK, P002). Extraction of nuclear proteins was performed via ExKine™ Nuclear Protein Extraction Kit (Abbkine, Wuhan, China, KTP3002). Then the protein samples were transferred to PVDF membranes (0.2 µm, Millipore, Burlington, MA, USA, ISEQ00010) after being separated by SDS-PAGE electrophoresis. The membranes were sealed by 5% defatted milk powder and then blotted with primary antibodies against Ki67 (1:1000, Abcam, Cambridge, UK, ab16667), p-H3 (1:5000, Abcam, ab32107), PCNA (1:2000, Proteintech, Wuhan, China, 10205-2-AP), Aurora B (1:1000, Abcam, ab287960), hexokinase II (HK2, 1:1000, Abcam, ab209847), glucose transporter 1 (GLUT1, 1:5000, Abcam, ab115730), LDHA (1:2000, Proteintech, 19987-1-AP), β-actin (1:5000, Abways, Shanghai, China, AB0035), and H3 (1:2000, Proteintech, 17168-1-AP) overnight at 4 °C. After combination with the corresponding secondary antibodies, the bands were detected with a gel imaging system (Amersham Imager 600, GE Healthcare Life Sciences, Marlborough, MA, USA).

### 2.5. Real-Time PCR Analysis

Total RNA was isolated from the treated primary mouse cardiomyocytes via VeZol Reagent (Nanjing, China, Vazyme, R411). After following the manufacturer’s protocol for HisyGo RT Red SuperMix (Vazyme, RT101) to synthesize cDNA from total RNA, then PCR product amplification was performed using the Roche LightCycler^®^ 96 System (Roche, Basel, Switzerland) with ChamQ Universal SYBR qPCR Master Mix (Vazyme, Q711). The primers used for the PCR analysis were as follows: nuclear factor erythroid-derived 2-like 2 (NRF2), forward 5’-GCC ACC GCC AGG ACT ACA G-3’ and reverse 5’-AAC TTG TAC CGC CTC GTC TGG-3’, SOD1, forward 5’-GCG GTG TGC GTG CTG AAG-3’ and reverse 5’-TCC TGA CAA CAC AAC TGG TTC AC-3’, CAT, forward 5’-CGG CAC ATG AAT GGC TAT GGA TC-3’, and reverse 5’-CTG GTC GGT CTT GTA ATG GAA CTT G-3’.

### 2.6. Dihydroethidium (DHE) Staining

DHE (10 μM, MCE, HY-D0079) is a peroxide indicator, which can penetrate cell membranes to detect intracellular ROS production. After being washed with PBS twice, treated cardiomyocytes or frozen sections (5 μm) were incubated with freshly prepared DHE solution for 30 min in the dark. The cardiomyocytes were washed with PBS twice again and then stained with Hoechst 33342 (a live nuclear marker dye, 10 μg/mL, MCE, HY-15559) for 10 min at room temperature. Finally, the stained cardiomyocytes were observed under fluorescence microscopy. DHE specifically reacts with intracellular superoxide anions and turns into ethidium in nuclei with red fluorescence, and the ratio of red fluorescence intensity to the basal level was considered to be the intracellular ROS level.

### 2.7. IF Staining of Cardiomyocytes

Cardiomyocytes were seeded on a confocal dish with a glass bottom and were treated differently according to the experimental design. Cells were fixed with 4% PFA for 20 min at room temperature, then blocked and permeabilized simultaneously with PBS containing 3% bovine serum albumin (BSA) and 0.1% Triton X-100 for 60 min. To detect cardiomyocytes specifically, primary cell samples were incubated with anti-cTnT antibody (1:400, Abcam, ab8295). For the cell proliferation assay, anti-Ki67 antibody (1:250, Abcam, ab16667) and anti-p-H3 antibody (1:400, Abcam, ab32107) were used to suggest that cardiomyocytes re-enter the cell cycle. EDU (10 μM, Beyotime Biotechnology, Shanghai, China, C0071S), which identifies DNA replication, was added to the medium 2 h before the end of treatment. The nuclear translocation of LDHA in cardiomyocytes was probed by the anti-LDHA antibody (1:200, Proteintech, 19987-1-AP). After a further incubation at room temperature for 1 h with corresponding fluorescent secondary antibodies, nuclear DNA was labelled in blue with DAPI. EDU staining requires additional incubation with the click reaction buffer for 30 min. Finally, cardiomyocytes were viewed under fluorescence microscopy. Images were analyzed using Image J (1.51j8, Madison, WI, USA). Specifically, nuclei were delineated by applying “Freehand Selection” to the DAPI signal. The corresponding cytoplasmic region for each cardiomyocyte was defined as the area within the cTnT-positive boundary excluding the segmented nuclear region. The fluorescence intensity of LDHA within each segmented nuclear region and its corresponding cytoplasmic region was measured via “Integrated Density, Area, and Limit to Threshold”.

### 2.8. Reduced Glutathione (GSH)/Oxidized Glutathione (GSSG)

GSH is a natural antioxidant in cells that can remove excess ROS produced during cell metabolism. GSSG is the oxidized form of GSH. The GSH/GSSG ratio is often used to reflect the level of oxidative stress in cells and tissues. The quantitative analysis of GSH and GSSG in cardiomyocytes is carried out following the instructions of the GSH content detection kit (Solarbio, Beijing, China, BC1175) and GSSG content detection kit (Solarbio, BC1185), respectively. Briefly, 5 × 10^6^ cells were lysed into 1 mL extracting solution via ultrasonic wave in an ice bath. Centrifuge 8000 g for 10 min, transfer the supernatant (usually 800 µL) for further measurement. Prepare the measuring tube, standard tube, and blank tube step by step according to the guides. Then, the absorbance of each tube at 412 nm was measured by a microplate photometer (Thermo, Waltham, MA, USA, 1410101). Finally, the content of GSH or GSSG is calculated according to the standard curve and the formula. The higher the ratio of GSH/GSSG, the lower the level of oxidative stress.

### 2.9. Statistical Analysis

All data were presented as the mean ± SEM from at least three independent experiments. Differences between two groups were analyzed with Student’s *t*-test. One-way ANOVA was used to analyze the multiple groups. The experimental data were analyzed using GraphPad Prism 7.0. *p* ≤ 0.05 was considered statistically significant.

## 3. Results

### 3.1. Nuclear LDHA Is Increased in the Remaining Cardiomyocytes After MI Injury

**Figure 1:**
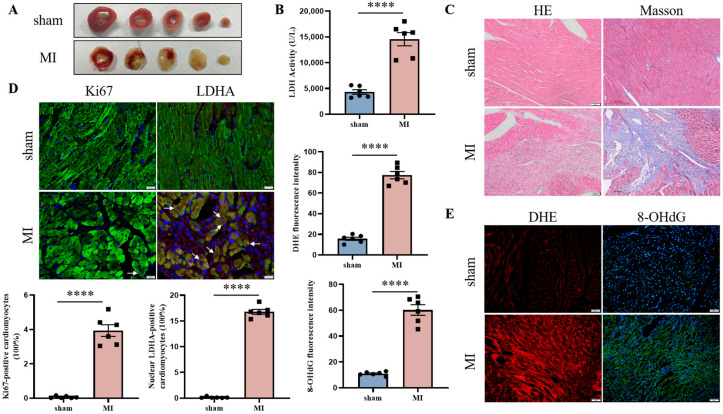
Nuclear LDHA is increased in the remaining cardiomyocytes after MI injury. C57BL/6 J mice MI models were constructed via permanent ligation of the left anterior descending branch. TTC staining (**A**) and serum LDH detection (**B**) were performed to verify cardiac damage. Heart cross-sections were stained with Masson’s trichrome (scale bar = 200 µm) to examine fibrosis and stained with H&E (scale bar = 50 µm) to detect the infiltration of inflammatory cells (**C**). IF staining of Ki67 (red, scale bar = 50 µm) and LDHA (red, scale bar = 20 µm) were performed to observe the cell cycle re-entry of cardiomyocytes (green) and nuclear translocation of LDHA in the infarct boundary region after MI injury, and white arrows indicate double-stained positive cardiomyocytes (**D**). The level of ROS production and oxidative damage to DNA were detected by DHE staining (scale bar = 50 µm) and 8-OHdG fluorescence probe (scale bar = 50 µm), respectively (**E**). Data are expressed as mean ± SEM, and compared by Student’s *t*-test. **** *p* < 0.0001 (*n* = 6).

Firstly, we constructed a mouse MI model via permanent ligation of the left anterior descending branch. TTC staining (Figure 1A) and serum LDH detection (Figure 1B) were performed 24 h after MI injury to prove that the mouse MI model was constructed successfully. Seven days after MI, Masson staining showed the formation of cardiac fibrosis, while H&E staining detected the infiltration of inflammatory cells (Figure 1C). These further confirmed the cardiac damage and repair after MI injury. Furthermore, there was a significant increase in the number of cardiomyocytes re-entering the cell cycle in the infarct boundary region, which was verified by the IF staining of Ki67 and cTnT. The nuclear translocation of LDHA in cardiomyocytes was also observed by the IF staining of LDHA (Figure 1D). Considering the fact that nuclear LDHA is always accompanied by oxidative stress in proliferative cells, we further assessed the level of oxidative damage in cardiac tissue after MI injury. Increased production of ROS was observed in the MI group by DHE staining, and strong oxidative damage to DNA was detected via the 8-OHdG fluorescence probe (Figure 1E).

### 3.2. Effects of Hypoxia on Cardiomyocyte Proliferation and Glycolysis In Vitro

**Figure 2:**
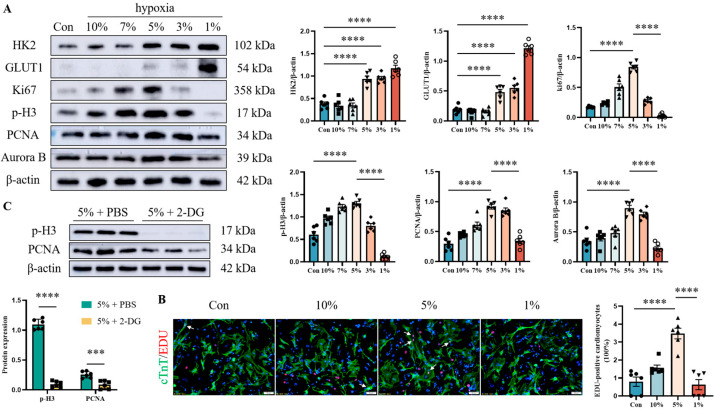
Effects of hypoxia on cardiomyocyte proliferation and glycolysis in vitro. Primary mouse cardiomyocytes were obtained from C57BL/6 J mice (1–2 days). Cardiomyocytes were exposed to hypoxia (10%, 7%, 5%, 3%, and 1%) for 12 h. The effects of hypoxia on glycolysis were evaluated by western blot analysis of GLUT1 and HK2, while Ki67, p-H3, Aurora B, and PCNA were used to evaluate the proliferation potential of cardiomyocytes. Histograms represent protein ratios normalized to β-actin (**A**). Double IF staining of cTnT and EDU further confirmed cardiomyocyte cell cycle re-entry (scale bar = 50 µm), and white arrows indicate double-stained positive cardiomyocytes (**B**). For glycolysis inhibition, cells were incubated with 2-DG (5 mM). The western blot detected the indicators of cardiomyocyte proliferation (p-H3 and PCNA) under 5% oxygen concentration along with 2-DG treatment. Histograms represent protein ratios normalized to β-actin (**C**). Data are expressed as mean ± SEM. Differences between two groups were analyzed with Student’s *t*-test. One-way ANOVA was used to analyze the multiple groups. *** *p* < 0.001, **** *p* < 0.0001 (*n* = 6).

To assess whether the cell cycle re-entry of cardiomyocytes in the infarct boundary region was a consequence of metabolic reprogramming induced by hypoxia, we determined the effects of hypoxia (10%, 7%, 5%, 3%, and 1%) on cardiomyocyte proliferation and glycolysis in vitro. There is no doubt that the level of glycolysis (HK2, GLUT1) increased with the decrease of oxygen concentration, which was significant at 1% oxygen concentration. Although severe hypoxia forced the metabolic switch to glycolysis, it failed to induce cardiomyocyte proliferation. From the western blot analysis of Ki67, p-H3, Aurora B, and PCNA, mild hypoxia (5%) motivated cardiomyocyte proliferation, while severe hypoxia (1%) induced cardiomyocyte cell cycle arrest (Figure 2A). The results of double IF staining (cTnT and EDU) further confirmed this phenomenon (Figure 2B). 2-DG is a glucose analog that acts as an inhibitor of glycolysis. To show whether glycolysis is essential for cardiomyocyte proliferation induced by mild hypoxia, we repeated the above experiments by employing 2-DG along with mild hypoxia treatment. The result of the western blot verified that indicators of cardiomyocyte proliferation (p-H3 and PCNA) were attenuated by inhibition of glycolysis (Figure 2C).

### 3.3. Nuclear LDHA Is Upregulated in Response to Severe Hypoxia-Induced Oxidative Damage of Cardiomyocytes

**Figure 3:**
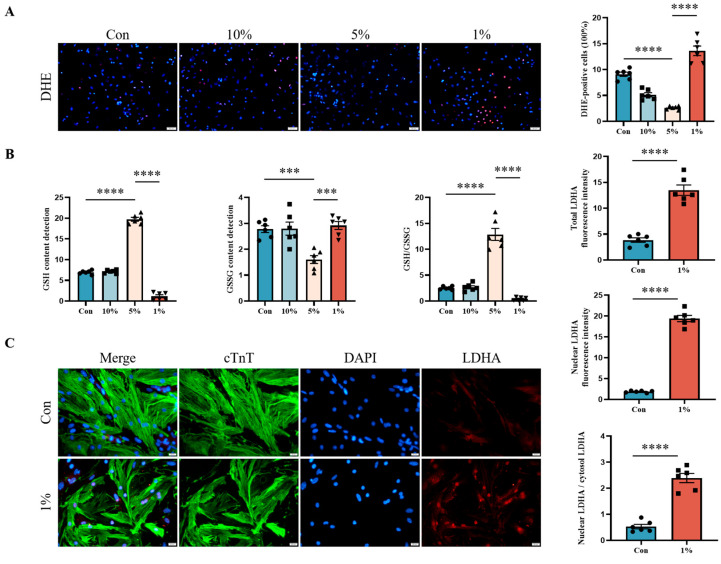
Nuclear LDHA is upregulated in response to severe hypoxia-induced oxidative damage of cardiomyocytes. Cardiomyocytes were exposed to hypoxia (10%, 5%, and 1%) for 12 h. ROS production in cardiomyocytes was detected by DHE staining (scale bar = 50 µm) (**A**). The redox state in cardiomyocytes under hypoxia was assessed via the GSH/GSSG ratio (**B**). The localization and quantification of LDHA in cardiomyocytes were detected by double IF staining of cTnT and LDHA (scale bar = 20 µm) (**C**). Data are expressed as mean ± SEM. Differences between two groups were analyzed with Student’s *t*-test. One-way ANOVA was used to analyze the multiple groups. *** *p* < 0.001, **** *p* < 0.0001 (*n* = 6).

It has been known that severe hypoxia damages the ETC, which leads to the production of ROS. We hypothesized that oxidative damage could be a significant factor contributing to cardiomyocyte cell cycle arrest under 1% oxygen concentration. Indeed, mild hypoxia (5%) alleviated the production of ROS in cardiomyocytes, while severe hypoxia coincided with excessive ROS generation, which was observed by DHE staining (Figure 3A). To further confirm our findings, we assessed the redox state in cardiomyocytes under hypoxia via the GSH/GSSG ratio (Figure 3B). We found that mild hypoxia upregulated the content of GSH (a natural antioxidant); severe hypoxia, however, increased the production of GSSG (the oxidized form of GSH). Since LDHA nuclear translocation occurs in response to oxidative stress, the localization and quantification of LDHA in cardiomyocytes were detected by double IF staining (cTnT and LDHA). As shown in Figure 3C, LDHA was mainly distributed in the cytosol and rarely in the nucleus of resting cells. After the stimulation of severe hypoxia, the expression of LDHA in the nucleus was dramatically increased. It is speculated that LDHA nuclear translocation in cardiomyocytes may occur in response to oxidative stress induced by severe hypoxia.

### 3.4. NAC Treatment Promotes Cardiomyocyte Cell Cycle Re-Entry While Preventing LDHA Nuclear Translocation

**Figure 4:**
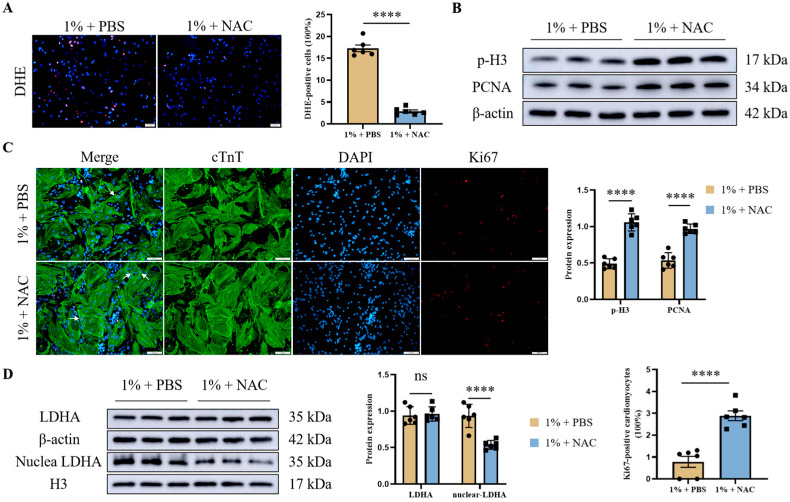
NAC treatment promotes cardiomyocyte cell cycle re-entry while preventing LDHA nuclear translocation. Cardiomyocytes were pre-treated with NAC (5 mM) for 4 h, and then exposed to 1% oxygen concentration for 12 h. ROS production in cardiomyocytes was detected by DHE staining (scale bar = 50 µm) (**A**). Western blot detected the indicators of cardiomyocyte proliferation (p-H3 and PCNA). Histograms represent protein ratios normalized to β-actin (**B**). Double IF staining of Cardiac Troponin T (cTnT) and Ki67 further confirmed cardiomyocyte cell cycle re-entry (scale bar = 100 µm), and white arrows indicate double-stained positive cardiomyocytes (**C**). The effects of NAC treatment on total LDHA and nuclear LDHA in cardiomyocytes under severe hypoxia were evaluated via western blot (**D**). Data are expressed as mean ± SEM, and compared by Student’s *t*-test. **** *p* < 0.0001, no significant difference (ns, *p* > 0.05) (*n* = 6).

To further determine whether oxidative damage is the cause of cardiomyocyte cell cycle arrest induced by severe hypoxia, primary mouse cardiomyocytes were pre-treated with NAC, a recognized inhibitor of ROS. After pre-treatment with NAC for 4 h, and then exposure to 1% oxygen concentration for 12 h, the strong fluorescence produced from DHE under severe hypoxia was obviously blocked (Figure 4A). Consistently, NAC treatment significantly restored the proliferative potential of primary mouse cardiomyocytes, which was verified by the western blot analysis of p-H3 and PCNA (Figure 4B). Double IF (cTnT and Ki67) staining of cardiomyocytes was performed to support the results of the western blot analysis (Figure 4C). Then we investigated the effects of NAC treatment on total LDHA and nuclear LDHA, respectively, in cardiomyocytes under severe hypoxia. There were no differences in the total LDHA expression level after NAC treatment of cardiomyocytes. Further extraction of nuclear proteins showed a rich level of LDHA in the nucleus of cardiomyocytes under 1% oxygen concentration, which was significantly down-regulated via NAC treatment (Figure 4D). Collectively, these data suggested that NAC treatment mitigates oxidative stress and is associated with enhanced cardiomyocyte proliferative marker expression, alongside a reduction in LDHA nuclear translocation under conditions of severe hypoxia.

### 3.5. ROS Represses Mild Hypoxia-Induced Cardiomyocyte Cell Cycle Re-Entry, Accompanied by LDHA Nuclear Translocation

**Figure 5:**
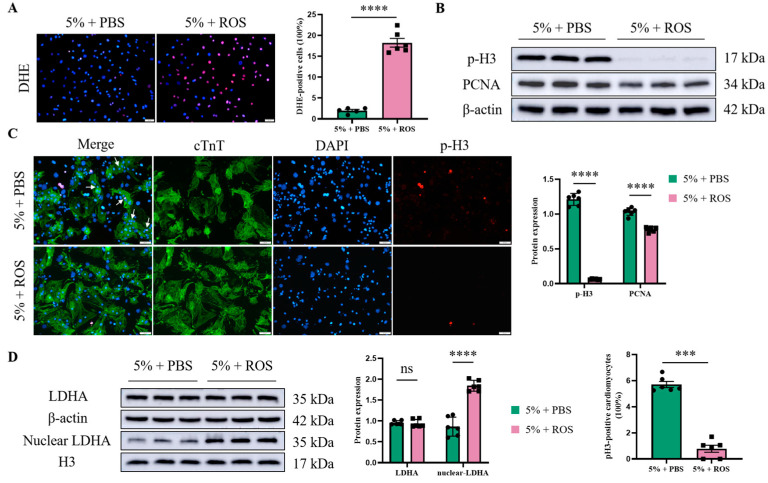
ROS represses mild hypoxia-induced cardiomyocyte cell cycle re-entry, accompanied by LDHA nuclear translocation. Berberine chloride is an alkaloid that induces ROS generation. Cardiomyocytes were pre-stimulated with Berberine chloride (10 µM) for 12 h, and then exposed to 5% oxygen concentration for another 12 h. ROS production in cardiomyocytes was detected by DHE staining (scale bar = 50 µm) (**A**). Western blot detected the indicators of cardiomyocyte proliferation (p-H3 and PCNA). Histograms represent protein ratios normalized to β-actin (**B**). Double IF staining of cTnT and p-H3 further confirmed cardiomyocyte cell cycle re-entry (scale bar = 50 µm), and white arrows indicate double-stained positive cardiomyocytes (**C**). The effects of ROS on total LDHA and nuclear LDHA in cardiomyocytes under mild hypoxia were evaluated via western blot (**D**). Data are expressed as mean ± SEM, and compared by Student’s *t*-test. *** *p* < 0.001, **** *p* < 0.0001, no significant difference (ns, *p* > 0.05) (*n* = 6).

As previously mentioned, mild hypoxia (5%) motivated cardiomyocyte cell cycle re-entry, along with the repression of ROS generation in cardiomyocytes. We tried to further investigate the effects of ROS on mild hypoxia-induced cardiomyocyte proliferation, as well as LDHA nuclear translocation. Herein, Berberine chloride (10 µM) is an alkaloid that induces ROS generation. The cardiomyocytes were pre-stimulated with Berberine chloride for 12 h, and then exposed to 5% oxygen concentration for another 12 h. Not surprisingly, the down-regulation of oxidative stress alleviated by mild hypoxia, which was observed by DHE staining, dramatically increased under the intervention of Berberine chloride (Figure 5A). Then we evaluated the effect of Berberine chloride on mild hypoxia-induced cardiomyocyte proliferation. From the western blot analysis of p-H3 and PCNA, we found that cardiomyocyte cell cycle re-entry mediated by mild hypoxia was significantly inhibited in the Berberine chloride group, where the cardiomyocytes suffered from severe oxidative damage (Figure 5B). Similar results were obtained from the double IF (cTnT and p-H3) staining of cardiomyocytes (Figure 5C). Moreover, the expression of total LDHA remained unchanged after the treatment of Berberine chloride, while nuclear LDHA was significantly upregulated in response to excessive ROS generated from Berberine chloride (Figure 5D). Taken together, these findings are consistent with a model where elevated oxidative stress is associated with reduced cardiomyocyte proliferative marker expression, but coincides with an increase in LDHA nuclear translocation.

### 3.6. Inhibition of LDHA Nuclear Translocation Aggravates Oxidative Stress and Cardiomyocyte Cell Cycle Arrest Under Severe Hypoxia

**Figure 6:**
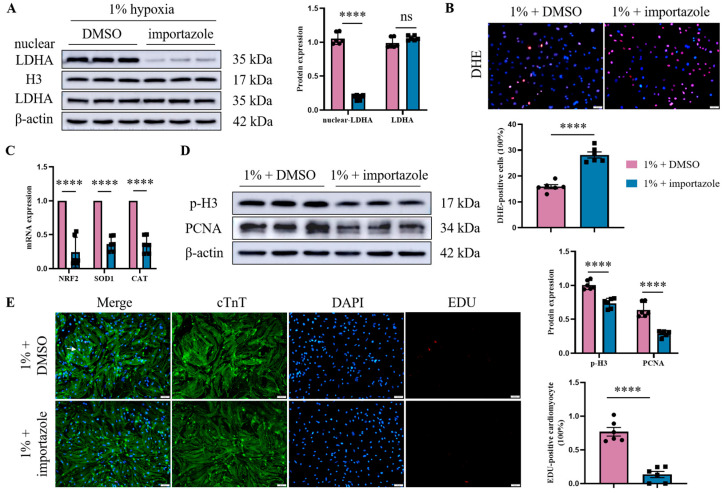
Inhibition of LDHA nuclear translocation aggravates oxidative stress and cardiomyocyte cell cycle arrest under severe hypoxia. For LDHA nuclear translocation inhibition, cardiomyocytes were pre-treated with importazole (40 µM) for 2 h, and then exposed to a 1% oxygen concentration for 12 h. Western blot confirmed the inhibition of LDHA nuclear translocation mediated by importazole (**A**). The effect of nuclear LDHA suppression on oxidative stress was detected by DHE staining (scale bar = 50 µm) (**B**). Real-time PCR analysis was performed to evaluate the expression of antioxidant genes (**C**). The western blot detected the indicators of cardiomyocyte proliferation (p-H3 and PCNA). Histograms represent protein ratios normalized to β-actin (**D**). Double IF staining of cTnT and EDU further confirmed cardiomyocyte cell cycle re-entry (scale bar = 50 µm), and white arrows indicate double-stained positive cardiomyocytes (**E**). Data are expressed as mean ± SEM, and compared by Student’s *t*-test. **** *p* < 0.0001, no significant difference (ns, *p* > 0.05) (*n* = 6).

Given our observations of increased nuclear LDHA expression both in the infarct area of MI in mice and in cardiomyocytes exposed to oxidative stress in vitro, we sought to explore the potential functional role of nuclear LDHA in modulating oxidative damage and cardiomyocyte cell cycle re-entry. We used importazole, a small molecule inhibitor of the nuclear transport receptor importin-β, to interfere with the nuclear translocation of LDHA in cardiomyocytes. In brief, the cardiomyocytes were incubated with importazole for 2 h, and then exposed to a 1% oxygen concentration for 12 h to construct a severe hypoxia model in vitro. Firstly, the inhibition of LDHA nuclear translocation mediated by importazole was confirmed via the western blot analysis of nuclear LDHA (Figure 6A). On this basis we further explored the effect of nuclear LDHA suppression on oxidative stress. Predictably, importazole treatment significantly increased ROS generation in cardiomyocytes, which was shown by a DHE fluorescent probe (Figure 6B). Furthermore, the expression of antioxidant genes (NRF2, SOD1, and CAT) was reduced by importazole treatment (Figure 6C). Finally, the proliferative potential of cardiomyocytes after importazole was detected via the western blot analysis (p-H3, PCNA) (Figure 6D) and the double IF (cTnT and EDU) staining (Figure 6E).

## 4. Discussion

Although LDHA nuclear translocation has been documented in certain proliferative cancer cells, where it can enhance antioxidant capacity and support survival under oxidative stress [15], its role in cardiomyocytes remains poorly understood. Previous work established that cytoplasmic LDHA promotes cardiomyocyte proliferation through metabolic reprogramming [13]. Our study provides novel evidence suggesting a potential non-canonical function for nuclear LDHA in cardiomyocytes, specifically related to redox homeostasis. Firstly, the expression of nuclear LDHA was induced both in the infarct area of MI in mice and hypoxic cardiomyocytes in vitro. Secondly, nuclear LDHA accumulation correlated with conditions of elevated oxidative stress in cardiomyocytes. Thirdly, pharmacological inhibition of LDHA nuclear translocation (using importazole) under severe hypoxia was associated with increased ROS, decreased antioxidant gene expression, and reduced cardiomyocyte proliferation. These findings suggested that nuclear LDHA translocation in cardiomyocytes may represent a compensatory response to oxidative stress, potentially contributing to the maintenance of redox balance and supporting cell cycle re-entry under ischemic conditions.

A sharp decrease in glycometabolic enzymes in the early postnatal window is the immediate cause of a shift from glycolytic to oxidative metabolism. Most glucose transporters and metabolic enzymes, including GLUT1, pyruvate kinase muscle isoenzyme 2 (Pkm2), and LDHA, decrease rapidly after birth [13,17,18,19]. Overexpression of metabolic enzymes (such as Pkm2 and LDHA) in cardiomyocytes promotes glucose flux into the pentose phosphate pathway (PPP) instead of mitochondrial oxidative phosphorylation, which reduces oxidative stress, ROS production, and oxidative DNA damage [13,18]. LDHA knockout neonatal mice exhibit damaged regeneration capacity after myocardial injury, while overexpression of LDHA in adult mice suffering from MI contributes to cardiomyocyte proliferation and repair [13]. It should be noted that besides the classical enzymatic function in the cytoplasm, LDHA translocates into the nucleus in response to excessive ROS, and nuclear LDHA gains a non-canonical enzymatic activity to produce antioxidant metabolites in cervical cancer cells [15]. Obviously, nuclear translocation of some glycometabolic enzymes, which are normally localized in the cytoplasm, may occur in response to certain cellular biological processes, such as cell division and proliferation. Indeed, our animal experimental results demonstrated increased nuclear LDHA in the remaining cardiomyocytes after MI injury. It has been reported that the number of cardiomyocytes re-entering the cell cycle in the infarct boundary region significantly increased in the adult mammalian heart after MI injury [20]. During the pathological state of MI, the cessation of oxygen supply compels a metabolic shift from oxidative phosphorylation to anaerobic glycolysis, which contributes to cell cycle re-entry of adult cardiomyocytes [21]. Herein, cell cycle re-entry of cardiomyocytes in the infarct boundary region after MI injury was accompanied by LDHA nuclear translocation, suggesting that the mechanism of LDHA nuclear translocation is potentially ubiquitous in proliferative cells.

Although gradual exposure to systemic hypoxemia, where inspired oxygen is gradually decreased to 7% and maintained for two weeks, reduces ROS and oxidative DNA damage, thereby causing cell cycle re-entry of cardiomyocytes in adult mice [6], the effects of hypoxia on cardiomyocyte proliferation is still controversial. Evidence shows that severe newborn anoxia, where ambient oxygen is <0.2% and is maintained for 10 min, accelerates cardiomyocyte terminal differentiation of newborn rats and results in a significant reduction in the number of cardiomyocytes [22]. It seems that the degree of hypoxia, in other words the concentration of oxygen, may be a main cause of this controversy. Sun Y et al. provide evidence that oxygen plays an indispensable role in the proliferation of cardiomyocytes in vitro, and neonatal cardiomyocytes cultured under hypoxic conditions (10% O_2_) for 7 days regained proliferative potential [23]. Our study further explored the effects of different oxygen concentrations on cardiomyocyte proliferation on the basis of hypoxia stimulation for 12 h. Moderate hypoxia (10%, 7%, and 5%) is indeed beneficial to promote cardiomyocyte cell cycle re-entry, which was significant at 5% oxygen concentration. There is no doubt that the level of glycolysis increased with the decrease of oxygen concentration. It is generally believed that glycolysis has a positive effect on cardiomyocyte proliferation, while severe hypoxia (1%) with remarkable glycolysis failed to induce cell cycle re-entry of cardiomyocytes. Although glycolysis is crucial for cardiomyocyte proliferation, cellular redox balance also plays an essential role [24]. An important finding of this study is that mild hypoxia (5%) prompted metabolic reprogramming and alleviated ROS, which was potentially linked to the cell cycle re-entry of cardiomyocytes, while severe hypoxia (1%) coincided with oxidative stress that induced cardiomyocyte cell cycle arrest.

As previously mentioned, nuclear LDHA acts as a sensor for overloaded ROS in cancer cells, which promotes antioxidant capacity, leading to cell survival and proliferation under oxidative stress. Mechanically, ROS-induced nuclear translocation of LDHA, accompanied with its tetramer-to-dimer transition, confers LDHA with a noncanonical enzyme activity to produce α-hydroxybutyrate (α-HB). α-HB accumulation caused a significant upregulation of histone H3K79 methylation by promoting the interaction between methyl-transferase and nuclear LDHA, resulting in the activation of antioxidant responses and the Wnt signaling pathway [15]. Given the high proliferation rate and the high production of ROS in cancer cells, LDHA nuclear translocation is an effective way to balance aberrant proliferation and oxidative stress injury. The disruption of the oxygen supply causes cardiomyocytes to suffer from severe hypoxia in the infarct area after MI injury, accompanied by grievous mitochondrial ETC damage and excessive free radical production [25,26,27]. Despite the metabolic status of mild hypoxia, hyperglycolysis, and low ROS generation in cardiomyocytes located in the infarct boundary region, the cell cycle re-entry is still threatened by massive free radical production in the infarct area. In this study, we found that nuclear LDHA accumulation correlated with conditions of elevated oxidative stress in cardiomyocytes, such as severe hypoxia or ischemia. Further investigation revealed that pharmacological inhibition of LDHA nuclear translocation under oxidative stress was associated with increased ROS and cardiomyocyte cell cycle arrest. This suggests a potential functional role for nuclear LDHA in mitigating oxidative stress and supporting cell cycle re-entry in stressed cardiomyocytes, potentially analogous to its role in cancer survival. However, cardiomyocytes are terminally differentiated cells with fundamentally different proliferative capacities and epigenetic landscapes compared to cancer cells. Furthermore, the sustained, dysregulated proliferation in cancer driving ROS production contrasts with the transient, controlled proliferative response sought in cardiac repair. Therefore, while the cancer model provides a valuable mechanistic hypothesis, the specific nuclear signaling role of LDHA in cardiomyocytes warrants dedicated investigation. Future studies should determine if α-HB production and H3K79 methylation occur in nuclear LDHA-positive cardiomyocytes, and, crucially, whether they are functionally required for the observed antioxidant or pro-proliferative effects in cardiomyocytes. NRF2, a transcription factor that regulates many cellular antioxidant/detoxification genes (such as SOD1 and CAT) [28,29,30], is involved in nuclear LDHA-mediated overcoming of oxidative stress. Currently, the data available in this study are not sufficient to elucidate the exact mechanism by which nuclear LDHA regulates the antioxidant response. Furthermore, given that importazole is a non-specific inhibitor of nuclear transporters, it is difficult to attribute its observed effects solely to the translocation of LDHA, as opposed to off-target effects on other proteins. Complementary strategies such as nuclear-targeted LDHA mutants need to be employed in future research.

## 5. Conclusions

Our study reveals the potential regulatory role of nuclear LDHA in resisting cardiomyocyte cell cycle arrest against oxidative stress injury. Mild hypoxia was associated with reduced ROS and cell cycle re-entry of cardiomyocytes, while severe hypoxia coincided with oxidative stress that induced cardiomyocyte cell cycle arrest. Notably, LDHA nuclear translocation in cardiomyocytes correlated with conditions of oxidative stress (e.g., severe hypoxia, ischemia). Pharmacological inhibition of this translocation was further associated with exacerbated oxidative stress and cardiomyocyte cell cycle arrest. These correlative observations lead us to hypothesize that nuclear LDHA accumulation may represent an adaptive response in cardiomyocytes, potentially contributing to redox balance and supporting the cell cycle re-entry of cardiomyocytes. Future studies should aim to elucidate the specific mechanisms of nuclear LDHA in cardiomyocytes and evaluate whether targeting nuclear LDHA serves as a promising strategy for cardiac repair in ischemic heart disease.

## Data Availability

The data are available from the corresponding author on reasonable request.

## Abbreviations

The following abbreviations are used in this manuscript:

LDHA	Lactate dehydrogenase A
MI	Myocardial infarction
ROS	Reactive oxygen species
ETC	Electron transport chain
2-DG	2-Deoxy-D-glucose
NAC	N-acetylcysteine
HK2	Hexokinase II
GLUT1	Glucose transporter 1s
NRF2	Nuclear factor erythroid-derived 2-like 2
DHE	Dihydroethidium
Pkm2	Pyruvate kinase muscle isoenzyme 2
PPP	Pentose phosphate pathway
